# Effect of whole-body vibration on freezing and flexibility in Parkinson’s disease—a pilot study

**DOI:** 10.1007/s10072-020-04884-7

**Published:** 2020-11-07

**Authors:** Andrea Dincher, Paula Becker, Georg Wydra

**Affiliations:** Sportwissenschaftliches Institut der Universität des Saarlandes, Saarbrücken, Germany

**Keywords:** Whole-body vibration (WBV), Parkinson’s disease (PD), Freezing, Flexibility

## Abstract

**Background:**

Parkinson’s disease is the second most common neurodegenerative disease. Symptoms are treated by medication, physio-, exercise, and occupational therapy. Alternative methods have been used in exercise therapy for a few years now. The effect of whole-body vibration as an alternative training method has been investigated for several symptoms in Parkinson’s disease. Since freezing and flexibility have not yet been investigated, the aim of this study was to evaluate the efficacy of different frequencies of application for these two symptoms.

**Methods:**

Patients were randomly assigned to a frequency (6, 12, or 18 Hz) or the control group. Before and after the treatment of 5 × 60 s with a rest of 60 s each, the Sit and Reach test (flexibility) and the 360° turn test (freezing) were performed.

**Results:**

Only the Sit and Reach test showed a significant improvement at 18 Hz (improvement from − 5.75 to − 1.89 cm, F(3,30) = 5.98**). At 360° turn, no significant differences were found. Weak to high effect sizes (standardized mean differences) were determined for the different frequencies, both for the Sit and Reach (from .01 to .64) and for the 360° turn (from − .72 to − 1.25). The highest effect size is observed for 18 Hz and the lowest for 6 Hz.

**Conclusions:**

Higher frequencies seem to be more effective than lower ones. Freezing, age, and gender also seem to play a role. Therefore, this should be investigated in further studies.

## Background

Parkinson’s disease is the second most common neurodegenerative disease [1]. The main symptoms of this disease include bradykinesia (slowing movement), hypokinesia (reduced movement amplitude and spontaneous movement) and akinesia (inhibition of movement initiation), rigor (muscle tone disorder, limited mobility) [2], tremor (trembling) [3], and postural instability (disorder of postural reflexes) [2]. Late motor symptoms include the on-off phenomenon after several years of treatment with dopamine medications, propulsion (tendency to fall forward) and freezing (involuntary blockage of movement) [4].

The symptoms are treated with medication, mainly to compensate for the dopaminergic deficit, with L-dopa products being most effective in combination with decarboxylase inhibitors. As the duration of treatment increases with the fluctuation of effect, MAO-B inhibitors (monooxidase-type B inhibitors) and COMT inhibitors (catechol-O-methyl transferase inhibitors) are prescribed as support. This results in a longer and more even duration of action of the L-dopa [5]. Deep brain stimulation can be mentioned here as an operative therapy measure [6]. A pulse generator (usually implanted below the clavicle) is implanted, which produces individually programmed electrical stimulation via the electrodes implanted in the subthalamic nucleus [5]. In addition, physiotherapy, ergo therapy, and speech therapy are usually prescribed for PD patients [5], which become more and more important as the duration of the disease increases, since here the medication-refractory symptoms such as freezing, gait and balance, and speech and swallowing problems occur more frequently [2]. An alternative treatment method in the field of physiotherapy is whole-body vibration (WBV). Mechanical vibrations are transmitted to the muscles via a platform on which the patient stands [7]. A distinction is made between harmonic and stochastic whole-body vibration, which can be induced on vertical or side-alternating plates [8]. The sinusoidal, harmonic whole-body vibration has the advantage that it can be used to test the effect of a certain frequency [9]. Only very few side effects are known, such as headaches or dizziness [10]. However, when standing on a vibration plate, these can be reduced or avoided by taking an upright, relaxed posture with slightly bent knees (approx. 26–30°) [10, 11]. However, there are contraindications such as acute thrombosis, inflammations, hernias, discopathies or rheumatoid arthritis, fresh bone fractures, or joint prostheses [12], but also cardiac arrhythmia, untreated hypertension, cardiac pacemakers, deep brain stimulators, aortic aneurysms, or migraines, which are also regarded as exclusion criteria for application frequencies above 15 Hz [13, 14].

In general, many positive effects of WBV have been reported that affect the strength [15] and flexibility [16] of different muscle groups or bone density [17] in both younger and older groups of people, whether athletes or non-athletes, or male or female. There are also positive effects in other diseases, e.g., cerebral palsy [18] or stroke [19]. In experiments with mice, WBV was shown to have a positive effect on brain function: improved balance beam performance and novel object recognition [20] and increased activity of the cholinergic system in the somatosensory cortex and amygdala [21]. These aspects are particularly important for PD patients. Treatment of symptoms with WBV in PD shows as many positive effects, especially in the main symptoms [22]. A single session of WBV compared to multiple sessions seem to lead to higher effects on main motor symptoms (SMD ± CI: .86 ± .03 for single session, .57 ± .05 for multiple session), especially for bradykinesia, tremor, and rigor. Mobility and balance were not investigated up to now for single session, only for multiple sessions, so it cannot be compared [23]. The effect of whole-body vibration on PD patients has been studied in recent decades on many aspects of symptomatology. There are many studies on balance, mobility, gait, and other motor symptoms such as tremor or bradykinesia, but with still inconsistent results due to different research methods (different application frequencies, frequencies, sentence numbers and lengths). The effect on freezing and mobility has not yet been investigated [24]. The effects of vibrations on freezing have so far only been investigated by the working group Winfree et al. [25] with partial vibrations via special shoes. A positive effect was achieved. Flexibility was also only investigated in a study with vibrating cuffs, with the result that the range of motion (ROM) improved in the hip joint, but not in the shoulder joint [26].

Thus, the present study will investigate the question of how different application frequencies of WBV affect freezing and flexibility in PD patients.

## Methods

The study was approved by the Ethics Committee of the Saarland University, application number 16-12 and was registered at the Deutsches Register Klinischer Studien (DRKS), registration number DRKS00012265.

### Sample of persons

The patients were recruited through medical practices, clinics, rehabilitation facilities, and self-help groups in Saarland and Rhineland-Palatinate, from January to April 2018. The study took place in the respective rooms of these organizations and at the Saarland University. Inclusion criteria, person suffering from PD, confirmed by a physician were included. Exclusion criteria, patients with the already described contraindications (e.g., fresh bone fracture/joint replacement, severe coronary heart disease, untreated high blood pressure, acute thrombosis, inflammations, hernias, discopathies, rheumatoid arthritis, cardiac arrhythmia, cardiac pacemaker, deep brain stimulator, aortic aneurism, or migraine) were not included according to the recommendations [12–14]. The sample consists of 36 persons, 50% male and 50% female. The average age is 69.29 ± 11.52 years, the average stage of disease according to Hoehn and Yahr is 2.11 ± .79, the subjects have been affected for an average of 7.36 ± 4.63 years, and the hip width is 33.25 ± 1.53 cm. There are 11 of the test persons suffer from freezing. Table 1 shows the characteristics of the sample sorted by test groups.

**Table 1:** Characteristics of the sample, sorted by test groups, means ± standard deviations

	Group 1	Group 2	Group 3	Group 4
Total number of subjects	9	9	9	9
Male	4	4	5	5
Female	5	5	4	4
Number of freezers	2	5	1	3
Age (years)	68.00 ± 9.09	70.70 ± 10.68	58.89 ± 10.11	77.33 ± 10.37
Duration of illness (years)	5.63 ± 5.66	8.50 ± 5.32	6.00 ± 4.82	6.00 ± 2.65
Disease stage (Hoehn & Yahr)	2.19 ± .70	2.11 ± .70	2.33 ± .90	2.11 ± .74
Hip width (cm)	32.83 ± 1.89	32.33 ± 1.21	34.67 ± 1.15	33.17 ± 1.60

When comparing the groups, only a significant age difference between group 3 and groups 2 and 4 can be observed.

### Variable sample

Freezing is examined with the 360° turn test, in which the patient rotates once to the right and once to the left around the body’s longitudinal axis. The number of steps required is measured [27]. In the 360° turn test, there is a trial in each direction. The number of steps per direction (360° turn left, 360° turn right) and the common mean value of both turn directions (360° turn combined) are evaluated. Flexibility is measured by the Sit and Reach test (S&R) [28]. The range is measured in centimeters while sitting (negative values above, positive values below the sole of the foot). The test person has one probation test and three main tests. The best value (best of 3) and the mean value of the three passes (mean of 3) are evaluated. Both test procedures were carried out directly before and directly after the treatment.

### Treatment sample

A side-alternating vibration platform (Galileo med Advanced) from Novotec Medical was used as the treatment. Three different vibration frequencies (6, 12, and 18 Hz) were used, and a placebo condition (control group, standing on the switched-off vibration plate) was created. The test persons were instructed to stand as upright and relaxed as possible with slightly bent knees (26–30°) without holding on to the platform, as recommended [9–11]. The stand width was set at 33 cm (average hip width of the sample) using adhesive tape on the panel. This corresponds to an amplitude of 4 mm. The test persons were not informed of which group they belonged to. For this reason, the display was concealed. The examiners were also blinded. Five sets of 60 s each were used with a 60 s pause between the sets with the corresponding frequency. The assignment to the different vibration frequencies was randomized by drawing lots showing only the group number, sorted by sex, so that the ratio within the groups should be balanced. Recruitment and assignment were done by A.D. and P.B..

Compared to multiple sessions, a single session of WBV leads to higher effects on motor symptoms. In addition, compared to randomized WBV, harmonic WBV leads to higher effects. Most of analyzed studies use a 5 × 60-s protocol that seems to lead to the highest effects [23], so all these parameters were chosen for this study.

**Fig 1:**
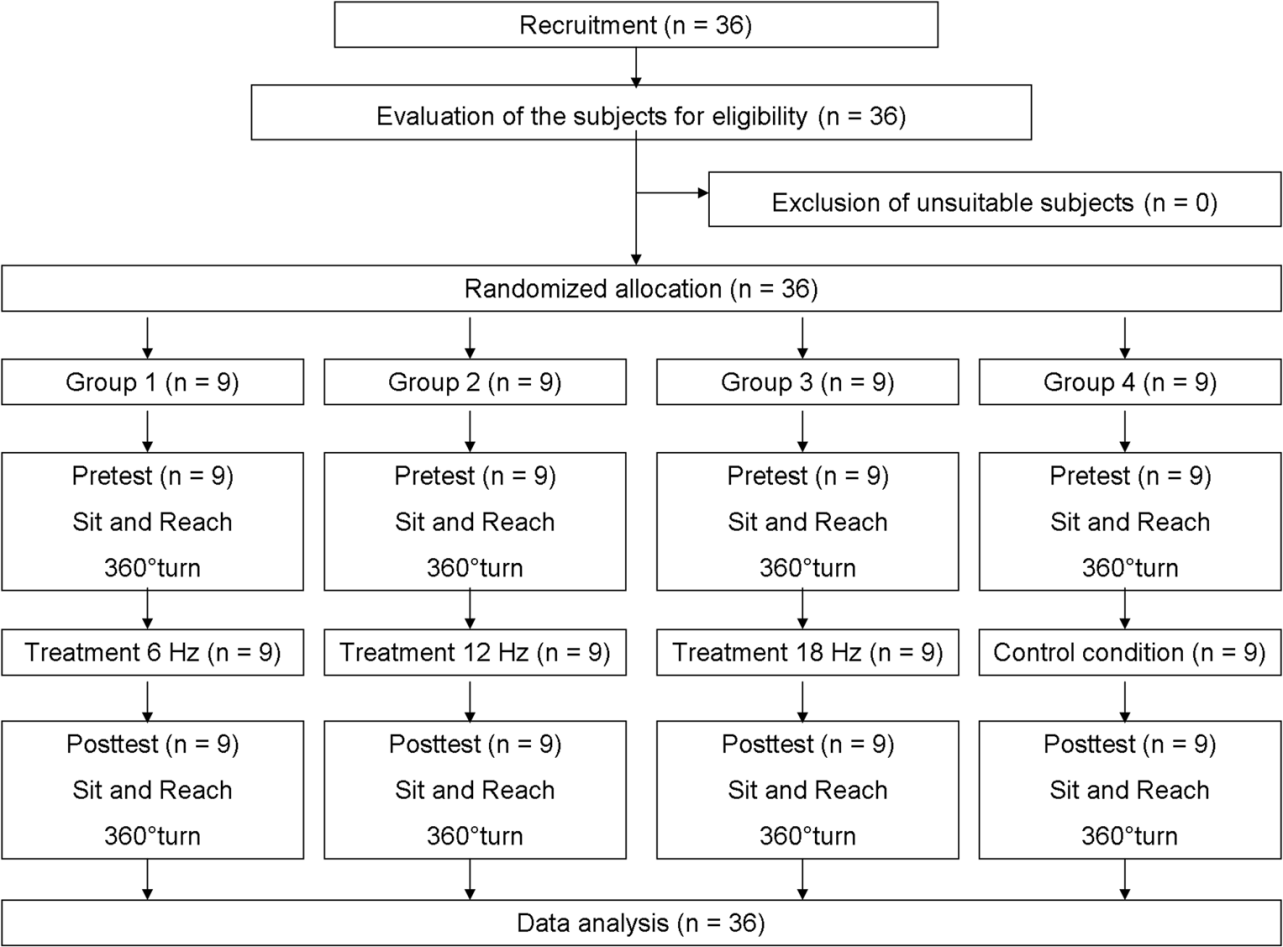
Flow diagram of the study process

Figure 1 shows the course of the examination.

### Hypothesis

There is a difference in performance between pre- and posttest freezing and mobility depending on the vibration frequency.

### Statistics

A K-S test was performed to verify a normal distribution of the data. In the case of a normal distribution, an ANOVA with measurement repetition is calculated; in the case of a non-normal distribution, a Kruskall-Wallis-ANOVA is calculated if the data do not suggest a normal distribution even after logarithms. The effects *time* (within, pre- to posttest), *frequency* (between, different application frequencies), and the interaction time*frequency are determined. Effect sizes are specified using standardized mean differences (SMD) and their 95% confidence interval (CI). The following categorization is performed: small effect SMD < .30, medium effect SMD > .50, large effect SMD > .80 [29].

Statistica 8 is used to calculate the differences and RevMan 5.3 is used to determine the effect sizes.

The significance level is set to *p* < .05.

## Results

**Fig 2:**
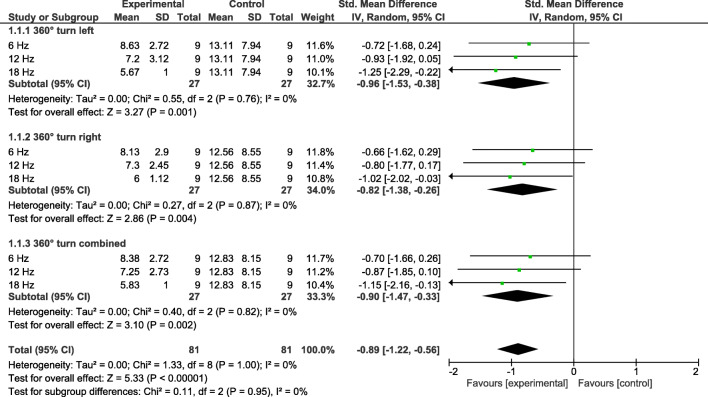
Results for 360° turn test (freezing), comparing experimental and control groups, using Standardized Mean Differences (SMD) and their 95 % confidence intervals (CI)

**Fig 3:**
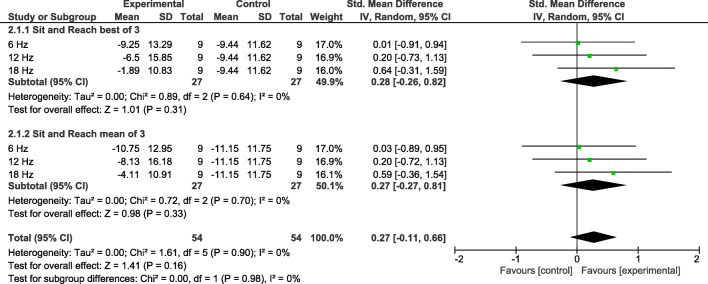
Results for Sit and Reach test (flexibility), comparing experimental and control groups, using Standardized Mean Differences (SMD) and their 95 % confidence intervals (CI)

A normal distribution of all variables is assumed. The following Table 2 gives an overview of the results of the pre- and posttests of the individual variables to compare the application frequencies. The following Figures 2 and 3 show forest plots concerning the effects sizes.

**Table 2:** Results of the pre- and post-tests for Sit and Reach and 360°turn for the test and control groups

	Sit&Reach best of 3	Sit&Reach mean of 3	360°turn left	360°turn right	360°turn combined
Group 1 (6 Hz)					
Pre	-11.00 ± 12.97	-12.08 ± 12.36	9.00 ± 3.25	8.50 ± 2.78	8.75 ± 2.96
Post	-9.25 ± 13.29	-10.75 ± 12.95	8.63 ± 2.72	8.13 ± 2.90	8.38 ± 2.72
Group 2 (12 Hz)					
Pre	-5.44 ± 15.34	-6.22 ± 15.40	7.50 ± 3.31	7.30 ± 3.56	7.40 ± 3.37
Post	-6.50 ± 15.85	-8.13 ± 16.18	7.20 ± 3.12	7.30 ± 2.45	7.25 ± 2.73
Group 3 (18 Hz)					
Pre	-5.75 ± 13.06	-6.75 ± 13.08	6.22 ± 1.48	6.44 ± 1.88	6.33 ± 1.62
Post	-1.89 ± 10.83	-4.11 ± 10.91	5.67 ± 1.00	6.00 ± 1.12	5.83 ± 1.00
Group 4 (control)					
Pre	-10.44 ± 8.22	-12.04 ± 8.77	12.11 ± 6.11	12.33 ± 7.30	12.22 ± 6.64
Post	-9.44 ± 11.62	-11.15 ± 11.75	13.11 ± 7.94	12.56 ± 8.55	12.83 ± 8.15

The ANOVA with repeated measurement (post hoc LSD) for the comparison of the different frequencies with the control condition shows the following results:

### Flexibility

#### S&R best of 3

Only factor *time* shows a significant result (F(3, 30) = 5.98**); factor *frequency* F(3, 30) = .47 and the interaction time* frequency F(3,30) = 1.32 are not significant. Only group 3 (18 Hz) shows a significant difference from pre- to posttest. Effect sizes (SMD ± 95 % CI) were determined for the 6 Hz-group .01 ± .92, for the 12 Hz-group .20 ± .93, and for the 18 Hz-group .64 ± .95.

#### S&R mean of 3

Neither factor *frequency* F(3, 30) = .47 nor factor *time* F(3, 30) = 2.48 nor the interaction time* frequency F(3, 30) = 1.27 show a significant result. Effect sizes (SMD ± 95 % CI) were determined for the 6 Hz-group .03 ± .92, for the 12 Hz-group .20 ± .93, and for the 18 Hz-group .59 ± .95.

### Freezing

#### 360° turn left

Only factor *frequency* shows a significant result (F(3, 30) = 4.38*); factor *time* F(3, 30) = .04 and the interaction time* frequency F(3, 30) = 1.52 are not significant. Effect sizes (SMD ± 95 %-CI) were determined for the 6 Hz-group .72 ± .96, for the 12 Hz-group − .93 ± .99, and for the 18 Hz-group − 1.25 ± 1.04.

#### 360° turn right

Only factor *frequency* shows a significant result (F(3, 30) = 3.52*); factor *time* F(3, 30) = .12 and the interaction time* frequency F(3, 30) = .13 are not significant. The following effect sizes (SMD ± 95 % CI) were determined for the 6 Hz-group − .66 ± .95, for the 12 Hz-group − .80 ± .97, and for the 18 Hz-group − 1.02 ± 1.00.

#### 360° turn combined

Only factor *frequency* shows a significant result (F(3, 30) = 3.99*); factor *time* F(3, 30) = .10 and the interaction time* frequency F(3, 30) = .57 are not significant. The following effect sizes (SMD ± 95 % CI) were determined for the 6 Hz-group − .70 ± .96, for the 12 Hz-group − .87 ± .97, and for the 18 Hz-group − 1.15 ± .98.

It should be noted that in the pretest, group 4 (control group) differed significantly from groups 2 and 3. In the posttest, all test groups differed significantly from the control group.

### Correlations

In addition, correlations were found between the age of the subjects and the performance in all variables from − .30 to .48**; the older the subjects were, the worse the performances were. Similarly, the relationships between the sex of the subjects and their performance were found in all variables from − .36* to .67***; on average, women performed better than men. A correlation between freezing of performance in sit and reach from − .36* to − .49** was also found; freezers performed worse than non-freezers. The stage and duration of the disease showed no correlation with the test results.

## Discussion

The purpose of this study was to investigate the effect of a single application of whole-body vibration on freezing and flexibility in PD patients. None of the volunteers reported a side effect. Some significant results were observed. In Sit and Reach (best of 3) a significant difference between pre- and posttest was found in the 18 Hz group with an effect size of .64. In 360° turn a significant group difference was found in the pretest. The effect sizes can be described as medium for the sit and reach for the 18 Hz group and weak to non-existent for the 6 Hz and 12 Hz groups. For 360° turn, the effect sizes are strong for the 12 Hz and 18 Hz group and medium for the 6 Hz group. It can therefore be assumed that higher frequencies are more effective than lower ones. This would be halfway consistent with Cardinale and Pope’s statement [30] that frequencies below 20 Hz have no effect, since the organs inside the body vibrate at a similar frequency [31] and these vibrations of muscles, bones, and joints must be constantly balanced [32]. Here it would be useful to test another frequency above 20 Hz in comparison to those investigated here. The assumption that frequencies below 15 Hz improve mobility [13] could not be confirmed here; in the 6 Hz group, only a slight improvement was observed, and in the 12 Hz group even a slight deterioration.

The partly insignificant differences in both assessments may be due to the unequal distribution of the freezers between the four groups. Second, the age of the subjects may have played a role. Group 3 (18 Hz) differs significantly from groups 2 (12 Hz) and 4 (control group). It can therefore be assumed here that younger people may benefit more from WBV application than elderly people. Another problem could be medication. All volunteers were tested in the ON state, at a time when medication is effective and symptoms are suppressed [4].

Looking at the pretest values at 360° turn, it is noticeable that, with the exception of group 4, all were within the norm value range corresponding to the Hoehn & Yahr stage (stage 2: 7.55 ± 1.96 steps, stage 2.5: 8.66 ± 2.66 steps) [23], and group 4 differed significantly from the others in the pretest. Nevertheless, all groups improved slightly with the exception of group 4, which deteriorated slightly. A placebo effect such as that described by Arias et al. [33] could not be confirmed here. The effect sizes of the 360° turn are considerably higher than those of the Sit and Reach, even at lower frequencies. It can be assumed that WBV has a better effect on freezing than on flexibility.

FOG is an episodic phenomenon and may disappear during the examination because the patient is paying extra attention to gait. The 360° turn might be not sensitive enough because of the lack of possible FOG triggering circumstances. FOG observation may require the use of triggering tricks, such as increased cognitive load (as in dual tasking), or stressful situations (as reacting under time pressure). So it would have been better to perform an additional assessment.

The Sit and Reach only measures the flexibility of hamstrings and hip joint. Besides, it leads to confounding factors such as the improvement of the rheological properties of the muscle after exercise, so it would have been better to perform an additional assessment.

Medium to high correlations between sex and performance in the 360° test were found. It confirms the findings of a number of studies that have reported the existence of gender difference on specific motor or non-motor symptoms in PD. Estrogen may play a protective role in PD by influencing dopamine synthesis and release or modulating dopamine receptor expression and function as seen in the analysis by Miller and Cronin-Golomb [34]

Pre- and posttest were only about 10 to 15 min apart, so it can be assumed that an exercise effect could have occurred in all groups, since there was a slight improvement in almost all groups and variables, partly also in the control group.

The sample may have been too small to be randomized; here it might have been more useful to match the test persons on the basis of freezing, sex, and age.

### Summary and prospects

The purpose of this study was to investigate the effect of a single WBV application on mobility and freezing in PD patients. It could be shown that higher frequencies seem to achieve a greater improvement from pretest to posttest than lower frequencies. However, the problem is that higher frequencies are not suitable for everyone because of the contraindications described. The study should therefore be tested with a larger sample matched to age, sex, and freezing in the OFF state using a further frequency above 20 Hz.
