# Correction to: Effect of whole-body vibration on freezing and flexibility in Parkinson’s disease—a pilot study

**DOI:** 10.1007/s10072-021-05464-z

**Published:** 2021-07-27

**Authors:** Andrea Dincher, Paula Becker, Georg Wydra

**Affiliations:** Sportwissenschaftliches Institut der Universität des Saarlandes, Saarbrücken, Germany

**Correction to** **:** **Neurological Sciences (2021) 42:2795–2801**

**https://doi.org/10.1007/s10072-020-04884-7**

The article “Effect of whole-body vibration on freezing and flexibility in Parkinson’s disease—a pilot study”, written by Andrea Dincher, Paula Becker, and Georg Wydra, was originally published Online First without Open Access. After publication in volume 42, issue 7, page 2795–2801 the author decided to opt for Open Choice and to make the article an Open Access publication. Therefore, the copyright of the article has been changed to © The Author(s) 2020 and the article is forthwith distributed under the terms of the Creative Commons Attribution 4.0 International License, which permits use, sharing, adaptation, distribution and reproduction in any medium or format, as long as you give appropriate credit to the original author(s) and the source, provide a link to the Creative Commons licence, and indicate if changes were made. The images or other third party material in this article are included in the article’s Creative Commons licence, unless indicated otherwise in a credit line to the material. If material is not included in the article’s Creative Commons licence and your intended use is not permitted by statutory regulation or exceeds the permitted use, you will need to obtain permission directly from the copyright holder. To view a copy of this licence, visit http://creativecommons.org/licenses/by/4.0.

The original article has been corrected.

